# Effects of Acute Normobaric Hypoxia on Memory Interference

**DOI:** 10.3390/brainsci9110323

**Published:** 2019-11-14

**Authors:** Paul D. Loprinzi, Aala’a Matalgah, Lindsay Crawford, Jane J. Yu, Zhaowei Kong, Bo Wang, Shijie Liu, Liye Zou

**Affiliations:** 1 Exercise and Memory Laboratory, Department of Health, Exercise Science and Recreation Management, The University of Mississippi, University, MS 38677, USA; pdloprin@olemiss.edu (P.D.L.); ammatalg@go.olemiss.edu (A.M.); lcrawfor@go.olemiss.edu (L.C.); 2 Exercise Psychology and Motor Learning Laboratory, Department of Sports Science and Physical Education, The Chinese University of Hong Kong, Hong Kong 999077, China; jieyu0203@gmail.com; 3 Faculty of Education, University of Macau, Av. da Universidade, Taipa, Macau 999078, China; zwkong@um.edu.mo; 4 Department of Psychology, Central University of Finance and Economics, Beijing 100081, China; wangbo@cufe.edu.cn; 5 Exercise and Mental Health Laboratory, Shenzhen Key Laboratory of Affective and Social Cognitive Science, Shenzhen University, Shenzhen 518060, China; liushijie0411@163.com

**Keywords:** BDNF, cognition, neuroprotection, hypoxia, normoxia

## Abstract

Purpose: Previous research has evaluated the effects of acute hypoxia exposure on cognitive function, notably executive function. No studies, to date, have evaluated the effects of acute hypoxia exposure on memory interference, which was the purpose of this experiment. Methods: A within-subjects, counterbalanced experimental design was employed, with condition (hypoxia vs. normoxia) and time (immediate vs. delayed) being the independent variables. Participants (*N* = 21; M*_age_* = 21.0 years) completed two laboratory visits, involving 30 min of exposure to either hypoxia (FIO_2_ = 0.12) or normoxia (FIO_2_ = 0.21). Following this, they completed a memory interference task (AB/AC paradigm), assessing immediate and delayed proactive and retroactive interference. Results: For retroactive interference, we observed a significant main effect for condition, *F*(1, 20) = 5.48, *p* = 0.03, ƞ^2^ = 0.10, condition by time interaction, *F*(1, 20) = 4.96, *p* = 0.03, ƞ^2^ = 0.01, but no main effect for time, *F*(1, 20) = 1.75, *p* = 0.20, ƞ^2^ = 0.004. Conclusion: Our results demonstrate that acute hypoxia exposure was facilitative in reducing memory interference. We discuss these findings in the context of the potential therapeutic effects of acute hypoxia exposure on synaptic plasticity.

## 1. Introduction

Various populations, such as mountaineers or mountain rescuers, may be at risk of cognitive dysfunction when ascending to higher altitudes. During states of acute hypoxia, turnover of key cognitive-related neurotransmitters (e.g., dopamine and norepinephrine) is reduced as a result of the oxygen requirement during the synthesis, release, and metabolism of these neurotransmitters [1,2]. Such alterations may, in theory, alter cognitive function. Narrative reviews indicate that acute and chronic hypoxia exposure may negatively influence several cognitive parameters, such as attention and executive function [3]. A recent meta-analysis demonstrated that acute hypoxia exposure (<60 mmHg for 10 min to 5 days) impairs both central executive and non-executive (e.g., memory) cognitive tasks [4]. For example, a recent experiment [5] demonstrated that acute (90 min) gradual exposure going from 20.9% O_2_ to 13.5% O_2_ (simulated altitude of 4500 m) impaired reaction time, but such an effect was attenuated with an acute (60 min; 50% of VO_2peak_) bout of exercise. Importantly, though, other related work [6] shows that under hypoxia (~30 min of simulated altitude of 3800 to 4500 m), exercise-induced improvements in cognition (reaction time) are attenuated for individuals exhibiting greater decreases in peripheral oxygen saturation.

We recently evaluated the effects of acute (30 min) moderate hypoxia (partial pressure of inspired oxygen (PIO_2_): 117 mmHg; fraction of inspired oxygen (FIO_2_): 0.154; altitude equivalent of 2500 m) on reaction time and central executive cognitive performance (Go/No-Go task) [7]. Participants (*N* = 20, M_age_ = 23.9 years) were exposed to a normoxic or hypoxic condition for 30 min, completed a pre-exercise cognitive task (Go/No-Go), then engaged in an acute bout of high-intensity interval exercise (or rest), and then re-completed the cognitive task immediately after exercise. Results demonstrated no moderate-hypoxia-induced impairment effects on reaction time or response accuracy [7]. Following this, we evaluated the effects of acute (10 min) severe hypoxia (PIO_2_: 87 mmHg; FIO_2_: 0.12; altitude equivalent of 4000 m) on central executive cognitive performance (Go/No-Go task) [8]. Participants (*N* = 30, M_age_ = 22.6 years) were exposed to the normoxic or hypoxic conditions for 10 min, completed the pre-exercise cognitive task, then engaged in an acute bout of moderate-intensity continuous exercise (or rest), and then re-completed the cognitive task during exercise. Our results demonstrated no severe-hypoxia-induced impairment effects on response accuracy [8]. Relatedly, other recent work (*N* = 15, M_age_ = 21.9 years) demonstrates that 30 min of severe acute hypoxia (equivalent to 4500 m) does not impair behavioral performance on a central executive cognitive task (i.e., Go/No-Go task), but does impair neural activity during inhibitory processing (i.e., reduced the peak amplitudes of Go- and No-Go-P300) [9].

Collectively, these studies suggest that short-duration (10–30 min) acute hypoxia may not be sufficient to induce behavioral impairments in central executive cognitive tasks. Although it is possible that the degree of hypoxia (i.e., altitude equivalent of 2500 m to 4500 m) was not enough to impair cognitive behavior (however, other work shows cognitive impairment effects at this altitude [5,6]), it is also possible that the cognitive tasks utilized in these studies were not sufficient to observe a hypoxia-induced impairment of cognition. That is, for these studies, the commission and omission error rates were very low. The present experiment addresses this latter issue. We aim to evaluate whether short duration (30 min) hypoxia at an altitude equivalent of 4000 m is sufficient to impair cognition. In order to enhance the sensitivity of the cognitive measure, instead of a central executive cognitive task, we will employ a paired-associative memory task that is highly sensitive to inducing error rates [10]. Further, paired-associative learning is dependent on the hippocampus [11], which is a brain structure particularly vulnerable to hypoxia [12]. The hippocampus also plays an important role in pattern separation and attenuation of memory interference [13]. Via this paired-associative task, we utilize an AB/AC memory interference paradigm that assesses both proactive and retroactive memory interference. Proactive memory interference involves the disruption of a subsequent memory from an earlier memory. In contrast, retrospective memory interference involves a subsequent memory disrupting the retrieval of a previously encoded memory.

Prior research has evaluated the effects of acute hypoxia on memory (not memory interference). Fowler et al. [14] evaluated the effects of acute hypoxia (arterial oxyhemoglobin saturation between 64%–66%) on short-term and long-term memory and found no evidence of a memory impairment effect. However, after 24 h of hypoxia exposure (simulated altitude of 4500 m), working memory was impaired [15]. Similar working memory impairments have been observed from shorter duration (30 min) acute hypoxia (5334 m or 7620 m) [16]. Although prior research has examined the effects of acute hypoxia on memory, no studies, to our knowledge, have evaluated the effects of acute hypoxia on memory interference specifically. This novel experiment is the first to evaluate the effects of acute hypoxia on immediate- and delayed-memory interference. Our a priori hypothesis was that acute hypoxia exposure would induce memory interference. The ultimate goal of this research was to establish this potential memory interference effect, and then in our subsequent research, evaluate the effects of other behaviors (e.g., acute exercise) on attenuating this potential hypoxia-induced memory interference effect.

## 2. Methods

### 2.1. Study Design

A within-subject, counterbalanced experimental design was employed. The experiment involved 2 visits, with each visit occurring approximately 24 h apart. Each participant completed both visits. Participants were blinded to the normoxia/hypoxia condition. Experimental procedures were approved by the ethics committee at the University of Mississippi and participants provided written informed consent prior to participation.

### 2.2. Participants

21 participants (10 males and 11 females) completed both visits. This was based on a power analysis indicating that 18 participants would be needed to achieve a power of 0.80, with inputs of 0.05 (α) and 0.08 (ƞ^2^). All participants were naïve, with no previous exposure to the normobaric procedure or exposure to laboratory-induced hypoxia. All participants lived at an approximate altitude of 154 m. Participants were excluded if they: (1) were outside the age range of 18–35 years, (2) exercised within 5 h of their visit, (3) consumed caffeine within 3 h of their visit, (4) were a daily smoker, (5) self-reported being pregnant, (6) had taken marijuana or other mind-altering substances in the previous 2 days, (7) had a diagnosis of attention deficit disorder/attention deficit hyperactivity disorder or took medications for these conditions, (8) consumed more than 1 alcoholic drink/day (female) or more than 2 alcoholic drinks/day (male), (9) had had a concussion in the past 30 days, (10) had a resting blood pressure ≥ 140/90 mmHg, (11) self-reported a previous episode of altitude sickness, (12) self-reported or diagnosed asthma, (13) were diagnosed with a neurological disorder, (14) measured body mass index > 35 kg/m^2^, or (15) had one or more of the following clotting risk factors: previously had a deep vein thrombosis (DVT), family history of DVT, previously had a thromboembolism, family history of thromboembolism, previous pulmonary embolism, family history of pulmonary embolism, Crohn’s disease diagnosis, currently taking anti-hypertensive medication, or previous orthopedic injury in the past 6 months.

### 2.3. Normoxia and Hypoxia

Similar to our previous work [7,8], normoxia and normobaric hypoxia was induced using the Hypoxic Training System (Everest Summit II Hypoxic Generator, New York, NY, USA). For these conditions, participants rested quietly in a seated position with a mask affixed over their mouth and nose for 30 min. The normoxic and hypoxic gas mixtures were generated through tubes that were connected to a breathing mask. The normoxic condition involved breathing a gas mixture involving FIO_2_ of 21%. The hypoxic condition involved breathing a gas mixture involving FIO_2_ of 12%.

Throughout this 30 min period, peripheral oxygen saturation (SpO_2_) was measured at 5 min intervals. The SpO_2_ was measured via a pulse oximeter (Zacurate, 500 BL) placed on the right index finger. During the 30 min period, and every five minutes, we also measured the participants’ perceptual responses (for safety reasons) using the 2018 Lake Louise Scoring System (LLSS) [17]. This questionnaire evaluates four symptoms (headache, gastrointestinal symptoms, fatigue/weakness, and dizziness/lightheadedness). Four items were assessed for each of these categories, with response options of 0 (no symptoms) to 3 (severe symptoms). The hypoxia exposure ceased if the total LLSS score was 3 or higher. Notably, no participants had a LLSS score of 3 or higher. Table 1 displays the schematic of the study protocol.

### 2.4. Memory Function

On a computer, participants completed a memory interference task using the AB/AC paradigm [19]. See Table 2 for an illustration of this paradigm. Participants were exposed to List 1 (AB–DE), which consisted of eight word pairs (e.g., Baby–Hunter) and then exposed to the Cued Recall List 1 where they recalled the missing word from the word pair (e.g., Baby–_____). Participants completed a 20 s distractor task (i.e., mathematical problems) and then were exposed to List 2 (AC–FG), which consisted of eight word pairs with some overlap of A words (e.g., Baby–Salad). After another 20 s distractor, participants were exposed to the Cued Recall of List 2 and attempted to recall the missing word from the word pair. Lastly, a final 20 s distractor was implemented and then participants completed the Modified Modified Free Recall (MMFR) word list where they recalled a combined list of List 1 and List 2. The MMFR consisted of all of the previously learned word pairs, with some have only one missing word (DE, FG) and others have two missing word pair associations (AB, AC) (e.g., Shoe–Movie, Baby–Hunter and Salad). After this MMFR, which was used to establish immediate memory interference, participants played on-line games for 20 min and then recompleted the MMFR for a delayed assessment of memory interference.

Using the participant’s MMFR results, the number of correctly recalled words from the subset of words (AB, DE are within List 1 and AC, FG are within List 2) were calculated. Using the cued recall results from List 1 and 2, proactive interference was measured by subtracting the percentage of correctly recalled FG pairs from the percentage of AC pairs (e.g., AC–FG) and retroactive interference was calculated by subtracting the percentage of correctly recalled DE pairs from the percentage of AB pairs (e.g., AB–DE). Higher interference scores indicate proactive and retroactive facilitation and thus correspond with decreased memory interference. That is, a lower interference score indicates a greater (worse, unfavorable) memory interference effect.

### 2.5. Statistical Analyses

JASP (vs. 0.10; The Netherlands) statistical software was utilized to analyze the data. A two-factor (condition and time) repeated measures ANOVA was employed. Specifically, a 2 (condition; normoxia vs. hypoxia) × 2 (time; immediate memory vs. delayed memory) repeated measures ANOVA was employed separately for proactive interference and retroactive interference. Statistical significance was set at an alpha lower than 0.05. Eta-squared (ƞ^2^) was calculated as an effect size estimate.

## 3. Results

Table 3 displays the characteristics of the sample. The sample was young (21.0 years), of near equal gender distribution (52.4% female) and heterogeneous regarding race-ethnicity (50.0% white).

**Figure 1:**
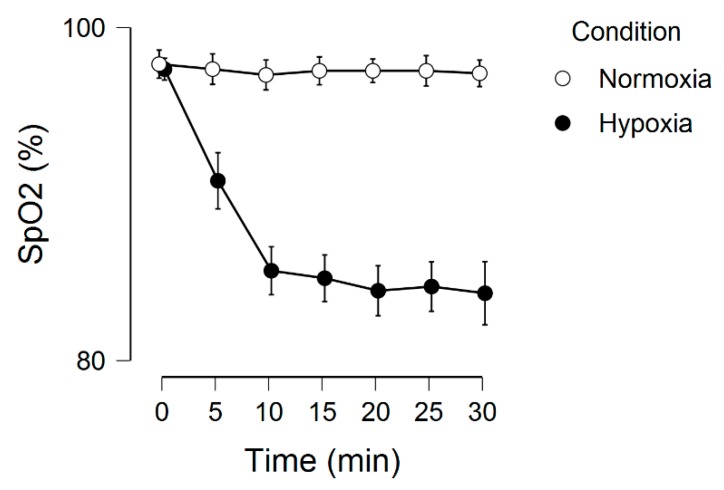
Peripheral oxygen saturation as a function of time and condition. Error bars represent 95% confidence intervals.

Figure 1 displays the SpO_2_ across the two experimental conditions. There was a significant main effect for condition, *F*(1, 19) = 239.0, *p* < 0.001, ƞ^2^ = 0.56, main effect for time, *F*(6, 114) = 54.8, *p* < 0.001, ƞ^2^ = 0.13, and condition by time interaction, *F*(6, 114) = 54.0, *p* < 0.001, ƞ^2^ = 0.11.

**Figure 2:**
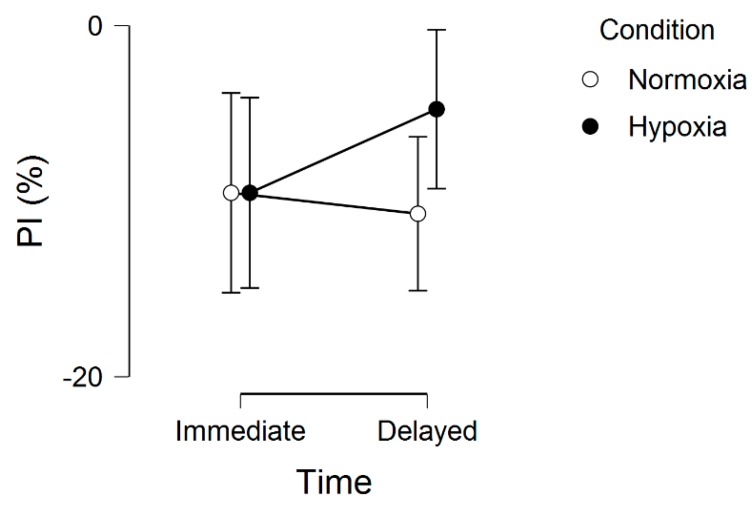
Proactive interference (PI) across normoxia and hypoxia for both the immediate and delayed memory assessments. Error bars represent standard errors.

**Figure 3:**
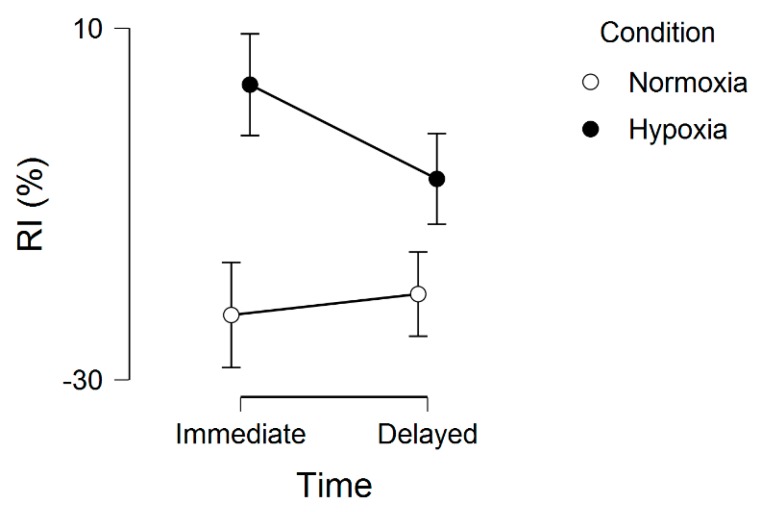
Retroactive interference (RI) across normoxia and hypoxia for both the immediate and delayed memory assessments. Error bars represent standard errors.

Regarding the memory interference outcomes, for proactive interference, there were no significant effects, including main effect for condition, *F*(1, 20) = 0.16, *p* = 0.69, ƞ^2^ = 0.002, main effect for time, *F*(1, 20) = 0.34, *p* = 0.56, ƞ^2^ = 0.001, and condition by time interaction, *F*(1, 20) = 0.74, *p* = 0.40, ƞ^2^ = 0.002. These results are graphically shown in Figure 2. For retroactive interference, we observed a significant main effect for condition, *F*(1, 20) = 5.48, *p* = 0.03, ƞ^2^ = 0.10, condition by time interaction, *F*(1, 20) = 4.96, *p* = 0.03, ƞ^2^ = 0.01, but no main effect for time, *F*(1, 20) = 1.75, *p* = 0.20, ƞ^2^ = 0.004. These results are graphically shown in Figure 3. Given that a lower interference score indicates a greater (worse, unfavorable) memory interference effect, these results demonstrate that the hypoxia condition had a higher score, suggesting a reduced memory interference effect.

## 4. Discussion

The purpose of this experiment was to evaluate the effects of normoxia/hypoxia on proactive and retroactive memory interference. The motivation for this experiment came from our earlier work evaluating the effects of hypoxia on other cognitive outcomes that did not reveal a detrimental effect. We anticipated that these null findings were a result of the cognitive measure used, and, as such, we employed a measure that we expected would be more sensitive to hypoxia. Further, the ultimate goal of our future work is to evaluate the extent to which acute exercise may attenuate hypoxia-induced memory function, including memory interference. Based on this, the present experiment was designed to first examine the extent to which hypoxia may induce memory interference. To our surprise, however, the result of the present experiment demonstrated that hypoxia induced retroactive memory facilitation, i.e., reduced retroactive memory interference. 

As stated, we anticipated that hypoxia would induce a memory impairment effect by specifically inducing memory interference. Unexpectedly, our results showed that hypoxia reduced a retroactive memory interference effect, with a trend towards also reducing a proactive interference effect. Upon careful consideration of this observation, we identified emerging research showing that, in certain situations, acute hypoxia may enhance brain function. This possibility has been thoroughly discussed elsewhere [20,21]. Other research has also shown that, for various conditions (e.g., metabolic syndrome), both normobaric and hypobaric hypoxia may have therapeutic effects [22,23]. 

The dose of hypoxia is what determines whether a therapeutic benefit or net pathological effect ensues. That is, low doses of acute hypoxia do not elicit detectable pathologies, such as hippocampal apoptosis or reactive gliosis [20]. Beneficial effects of a single exposure of acute hypoxia has occurred from intermittent protocols involving 3 to 15 episodes, 5 min in duration, with 5 min intervals (i.e., 15 min to 75 min of hypoxia exposure) [20]. This 15–75 min exposure aligns with our present experiment (i.e., 30 min of exposure), but, notably, we acknowledge that the physiological and neurochemical responses to intermittent hypoxia protocols may not be the same as continuous protocols [24,25]. However, two of the recently recognized unexpected benefits of low-dose acute hypoxia include improved respiratory and non-respiratory somatic motor function and increased growth factor expression in the central nervous system.

Increased growth factor production, including upregulation of brain-derived neurotrophic factor (BDNF), plays a critical role in subserving memory function [26]. Tropomyosin-regulated kinase B (TrkB), a BDNF receptor, is regulated by hypoxia-inducible factor-1, a transcription factor regulated by hypoxia-sensitive genes [27]. Thus, TrkB regulation is upregulated in the whole brain following acute hypoxia exposure. These acute effects may facilitate memory function, as BDNF has been shown to upregulate the expression and function of key memory-related receptors, namely NMDA [28,29,30]. Further, a key mechanistic correlate of episodic memory function is long-term potentiation, that is, sustained neurotransmitter release to maintain excitatory post-synaptic potentiation. Downstream of the BDNF/TrkB, pathway, PI3K-AKT activation helps maintain LTP via NMDA activity [31]. Thus, hypoxia-induced BDNF production may help facilitate memory via increasing synaptic strength (i.e., plasticity). In addition to BDNF-related improvements in episodic memory function, BDNF also appears to play a critical role in neuronal pattern separation of similar content [32], which has implications in reducing memory interference.

In conclusion, this experiment is the first to demonstrate that acute hypoxia exposure can reduce retroactive memory interference. If replicated, this has important implications on multiple levels. For example, it would be worthwhile to investigate whether acute hypoxia can attenuate memory interference among populations (e.g., older adults) that are vulnerable to memory interference. It would also be interesting to investigate whether acute hypoxia, coupled with other strategies thought to enhance memory (e.g., acute exercise), have a synergistic effect on enhancing memory and reducing memory interference. Such future work should aim to overcome the limitations of this experiment, which includes, for example, the relatively small, homogeneous sample, as well as the lack of a mechanistic measure (e.g., BDNF). Further, future work should more precisely and comprehensively evaluate the physiological responses to hypoxia exposure. For example, when feasible, more accurate measures of arterial oxygen saturation (via arterial blood assessment) should be evaluated. Further, there is evidence [33,34] to suggest that hypobaric and normobaric hypoxia environments may induce unique physiological responses (e.g., slightly different minute ventilation and nitric oxide levels), and, as such, future work may wish to evaluate whether the type of hypoxia exposure has a unique effect on memory interference. Relatedly, such work should carefully evaluate the rate of breathing during hypoxia exposure, as the rate and depth of breathing can reduce the partial pressure of arterial carbon dioxide (PaCO_2_). Such resultant hypocapnia effects may cause respiratory alkalosis and cerebral vasoconstriction, ultimately exacerbating cerebral hypoxia. The present experiment also did not measure water vapor pressure (P_H2O_), which may be an important parameter to evaluate, as it may influence the inspired partial pressure of oxygen. Such recommendations will be critical in future work that aims to evaluate the effects of specific doses of hypoxia on memory interference. 

## Figures and Tables

**Table 1:** Study protocol.

Normoxia (PIO_2_ = 150 mmHG, FIO_2_ = 0.21) ^†^				Normobaric Hypoxia (PIO_2_ = 87 mmHG, FIO_2_ = 0.12, simulated altitude of 4000 m) ^‡^			
30-min exposure ^†^	Memory Task	20-min rest *	Delayed Memory Recall	30-min exposure ^‡^	Memory Task	20-min rest *	Delayed Memory Recall

**^†^** The 30 min exposure of normoxia involved continuous breathing of FIO_2_ = 0.21. **^‡^** The 30 min exposure of acute hypoxia involved a gradual progression from FIO_2_ = 0.21 to FIO_2_ = 0.12 over the first 5 min. That is, within the first 5 min of acute hypoxia exposure, there was a gradual progression to an FIO_2_ = 0.12, and then from minutes 5–30, the FIO_2_ stayed at 0.12. During the 30-min normoxia/hypoxia exposure, participants watched a blooper video of the popular television show, The Office. * In order to prevent boredom, during the 20 min rest period, participants played on-line games [18].

**Table 2:** Example protocol for evaluating memory interference. MMFR, Modified Modified Free Recall.

Study Set 1: AB, DE	Cued Recall 1: A__, D__	Study Set 2: AC, FG	Cued Recall 2: A__, F__	MMFR: A__ __ D__ __ F__ __
**BABY HUNTER**	SPIDER ________	**FOREST CITY**	ARROW _______	**BABY** ____ ____
**SUPPER SHERIFF**	**FOREST** ________	ARROW THEATER	**FOREST** ______	CHERRY ____ ____
SHOE MOVIE	**BABY** ________	**BABY SALAD**	TIGER ______	ARROW ____ ____
SPIDER CANDLE	CHERRY ________	TIGER HOTEL	**SUPPER** _______	**SUPPER** ____ ____
**MONKEY GARDEN**	**MONKEY** ________	**MONKEY ENGINE**	LADY _______	TIGER ____ ____
**FOREST BATTLE**	**SUPPER** _______	LADY BUTTER	CANNON _______	SHOE ___ ___
CHERRY MONEY	APPLE ________	CANNON HAMMER	**BABY** _______	**MONKEY** ____ ____
APPLE DIAMOND	WEDDING ______	**SUPPER JACKET**	**MONKEY** ______	LADY ____ _____
				SPIDER ____ ____
				**FOREST** ____ ____
				APPLE ____ ____
				CANNON ____ ____

Bold words represent the interference pairing (AB/AC).

**Table 3:** Participant characteristics.

Variable	Point Estimate	SD
Age, mean years	21.0	2.1
% Female	52.4	
Race-Ethnicity, %		
Mexican American	4.5	
Other Hispanic	4.5	
Non-Hispanic white	50.0	
Non-Hispanic black	9.1	
Multi-race/other	31.8	
BMI, mean kg/m^2^	26.8	5.7
